# Search for RNA aptamers against non-structural protein of SARS-CoV-2: Design using molecular dynamics approach

**DOI:** 10.1186/s43088-021-00152-5

**Published:** 2021-10-12

**Authors:** Ram Kothandan, Pavithra Uthayasooriyan, Sivaranjani Vairamani

**Affiliations:** 1 Bioinformatics Laboratory, Department of Biotechnology, Kumaraguru College of Technology, Coimbatore, India; 2 Department of Biotechnology, Kumaraguru College of Technology, Coimbatore, India

**Keywords:** SARS-CoV-2, Aptamer, NSP10, Docking, Molecular dynamics

## Abstract

**Background:**

Recent outbreak of deadly Severe Acute Respiratory Syndrome Coronavirus-2 (SARS-CoV-2) urges the scientist to identify the potential vaccine or drug to control the disease. SARS-CoV-2 with its single stranded RNA genome (length ~ 30 kb) is enveloped with active spike proteins. The genome is non-segmental with 5’-cap and 3’-poly tail and acts as a mRNA for the synthesis of replicase polyproteins. The replicase gene lying downstream to 5’-end encodes for non-structural protein, which in turn pose multiple functions ranging from envelope to nucleocapsid development. This study aims to identify the highly stable, effective and less toxic single strand RNA-based aptamers against non-structural protein 10 (NSP10). NSP10 is the significant activator of methyltransferase enzymes (NSP14 and NSP16) in SARS-CoV-2. Inhibiting the activation of methyltransferase leads to partial viral RNA capping or lack of capping, which makes the virus particles susceptible to host defence system.

**Results:**

In this study, we focused on designing RNA aptamers through computational approach, docking of protein-aptamer followed by molecular dynamics simulation to perceive the binding stability of complex. Docking study reveals the high binding affinity of three aptamers namely RNA-053, 001, 010 to NSP10 with the HADDOCK score of − 88.5 ± 7.0, − 87.7 ± 11.5, − 86.1 ± 12 respectively. Molecular Dynamics suggests high conformational stability between the aptamer and the protein. Among the screened aptamers two aptamers maintained at least 3-4 intermolecular H-bonds throughout the simulation period.

**Conclusions:**

The study identifies the potential aptamer candidate against less investigated but significant antiviral target i.e., NSP10/NSP16 interface complex.

**Supplementary Information:**

The online version contains supplementary material available at 10.1186/s43088-021-00152-5.

## Background

At the end of 2019, the viral pneumonia similar to pneumonia was reported in Wuhan, Hubei, People’s Republic of China. After sample analysis the clinical features revealed that the pneumonia was caused by the novel coronavirus. In the early 2020, World Health Organization (WHO) and International Committee on Taxonomy of Viruses (ICTV) have named the disease as COVID-19 and the virus as Severe Acute Respiratory Syndrome Corona Virus-2 (SARS-CoV-2) [1, 2]. The highly transmissible SARS-CoV-2, part of the betacoronaviridae family possesses single stranded RNA genome. The length of CoV-2 genome is ~ 30 kb, known to be the largest RNA genome shares 82% of genome sequence identity with SARS-CoV. The prevalent indications of COVID-19 are headache, fever, runny nose, sore throat and shortness of breath but in serious conditions it leads to multiorgan failure, cytokine storm and septic shock [3, 4]. The earlier medication includes polymerase inhibitors such as remdesivir, favipiravir and repurposed drugs like chloroquine, hydroxychloroquine, arbidol and other therapies include convalescent plasma, immunoenhancement therapy. However, the repurposed drugs such as chloroquine and its derivatives are no longer used medicament against SARS-CoV-2. Due to low efficacy, adverse effects of drug candidates, expensiveness and safety concern of therapies there is a desperate need to develop a potent therapeutic treatment against COVID-19 [5].

The SARS-CoV-2 genome is organized into core genome and accessory genome. The core genome encodes for structural proteins and non-structural proteins are considered essential in viral genome production, replication, viral particles formation and its assembly [6, 7] whereas the accessory genome encoding proteins are responsible for its spread and virulence [8]. The polypeptides of ORF1ab gene are processed into 16 various non-structural proteins each having unique activities like host-immune response suppression, proteolysis, helicase and methyltransferase activity, RNA replication [9]. The non-structural protein 10 (NSP10) is the co-stimulatory factor of NSP14 and NSP16. These proteins are involved in methyl transferring activity i.e., viral RNA capping, in which the NSP10 plays a vital role by stabilizing the S-adenosyl-L-methionine (SAM), a methyl donor binding site. The NSP14 or N7-methyltransferase is actively involved in proofreading/exonuclease activity and viral RNA capping. Researchers have reported that the 2′OH-methyltransferase activity of NSP16 is an imperative mechanism for coronavirus replication in cell cultures [10].

As known, mRNA of eukaryotic cells undergoes post-transcriptional modifications like 5′-RNA capping, splicing and polyadenylation before being exported to cytoplasm. Similarly, these viruses utilize the RNA capping mechanism as a weapon to avoid the encounter by host defence mechanism, to improve RNA stability and efficient translation using its own RNA capping machinery [11]. The host and/or viral ribonucleic acid molecules are degraded in the cytoplasm if found without the RNA cap [12]. Thus, this mechanism might be considered as a promising antiviral target for developing the therapeutic drugs/treatment against COVID-19.

DNA/RNA aptamers are single stranded oligonucleotide with defined structure which binds to target specifically. They can be targeted against nucleic acids and protein molecules. The main advantages of aptamer are less immunogenic, easily synthesized or modified, long term storage and stable in various conditions like temperature or pH [13]. RNA aptamers are identified through the Systematic Evolution of Ligands EXponential enrichment (SELEX) method experimentally. The SELEX process involves library construction, binding, elution, amplification and repetition of mentioned steps with enriched oligonucleotide pool [14]. Multiple rounds of SELEX results in stringent aptamer against the target but this method is time consuming and expensive. To overcome this hurdle RNA aptamers can be screened computationally. The in-silico screening process involves the randomized RNA pool construction, secondary structure analysis, hybridization and optimization of potential aptamer candidate. At present, several researchers across the world manifested the use of aptamer-based medication against SARS-CoV-2 [15–17].

Current study focuses on emerging field of aptamer technology for the identification of specific RNA based aptamers to confront SARS-CoV-2 by targeting the NSP10. This work provides the structural insights of RNA aptamer and NSP10 interaction which are studied through molecular docking and molecular dynamics approach.

## Methods

### Structure retrieval

The 3D structure of most significant protein in SARS-CoV-2 viral capping mechanism i.e., NSP10 (PDB id: 6ZCT with resolution of 2.55 Å) was retrieved from RCSB Protein Data Bank (PDB) [18]. The crystal structure of NSP10-NSP16 complex with accession code of 6W75 (1.95 Å) was also downloaded from PDB to study about the interfacial residues involved in binding of NSP10–NSP16.

### In silico RNA aptamer design

The 30-mer RNA pool of 100 sequences were generated randomly using shuffleseq program of EMBOSS package [19]. The RNA sequences were screened on the basis of (1) GC content > 40% (2) the minimum free energy of RNA secondary structure lower than − 5.7 kcal/mol (3) Last three nucleotides of 5’ and 3’ end being complementary/binds with each other [20]. The screened aptamer sequences were subjected to 2-dimensional structure prediction by RNAFold of Vienna RNA software suite [21]. The dot bracket secondary structure notation of aptamers obtained from RNAfold was submitted in RNAComposer for 3D structure prediction [22].

### Protein-aptamer docking

Prior to docking, the water molecules and/or ions or small molecules found in retrieved structure of protein were eliminated and saved for further studies. The 3D form of protein and predicted RNA aptamers were energy minimised to obtain its stable conformation. The energy minimisation was carried out by GROMACS v2020.4 [23] using AMBER96 force field [24]. The steepest descent algorithm with maximum step size of 50,000 was used to energy minimize the molecules.

Protein- RNA aptamer docking was performed by High Ambiguity Driven biomolecular DOCKing (HADDOCK v2.2) web server [25]. This data driven method performs biomolecular complexes modelling by 3 major steps. The stages involved in docking are (1) rigid body energy minimization (2) semi-flexible refinement in torsional angle space (3) final refinement in explicit solvent. At each stage the resultant conformations are weighed/ scored and ranked, to find the best conformation of the complex. The HADDOCK score is weighed sum of van der Waals, electrostatic, desolvation and restraint violation energies together with buried surface area [25]. The interactions between NSP10 and each aptamer were visualized and analysed by PyMol and Ligplot + [26], Protein–Ligand Interaction Profiler [27]. All 3D protein and docked complexes illustrations were generated using Protein Imager, an online molecular viewer [28].

### Molecular dynamics simulation

To determine the binding efficacy and stability of the protein-RNA binding, molecular dynamics was performed using GROningen MAchine for Chemical Simulations (GROMACS 2020.4) [23]. Simulation was done for Apo-protein (NSP10) and the 3 protein-RNA Complex with the highest score obtained from docking analysis. The MD simulation was carried out for a period of 200 ns. The AMBER96 force field was used for the simulation of the systems [24]. Topology parameters were generated for Protein-Aptamer and Zinc (Zn) ion individually and then combined later during the actual simulation process. The systems were placed in dodecahedron box with the size of 1.0 nm and filled with TIP3P water model [29]. The box was neutralized by adding Na^+^ and Cl^−^ ions while maintaining the buffer concentration at 0.1 M NaCl. Steepest Descent Algorithm was used to energy minimize the built systems with maximum of 50,000 steps and with energy tolerance of 1000 kJmol^−1^ nm^−1^. Bond lengths in the system were constrained using LINCS algorithm [30] and periodic boundary constraints were applied in all XYZ directions. Long-range electrostatics in the systems with 0.16 nm Fourier spacing and 1.0 nm cut-off was computed by using the Particle Mesh Ewald method (PME) [31]. The V-rescale and Parrinello-Rahman coupling methods were employed for the equilibration of the NVT and NPT ensembles. In the NVT ensemble, a constant number of particles (N), volume (V) and temperature (T), a coupling constant of 0.1 ps for 1000 ps and a constant temperature of 300 K were used. In the NPT ensemble, a constant number of particles (N), pressure (P) and temperature (T), 1 bar as constant pressure and the same coupling constant parameter were used [32].

The equilibrated systems were set up for final production run for 200 ns. The structural coordinates were saved for every 10 ps and resultant trajectories were analyzed using GROMACS v2020.4 modules. Molecular Dynamics simulation were executed on an Intel i7 workstation powered by Nvidia GTX 2080Ti GPU system and analysis were carried out on Dell PowerEdge T20 workstation with 32 GB RAM.

### MD simulation analysis

The gmx rms, gmx rmsf, gmx gyrate and other important modules were used to study about the conformational stability, flexibility of residues and compactness of the protein and docked complexes. MD analysis was carried out as mentioned in our previous study [32].

The solvent accessible surface area (SASA) was calculated using gmx sasa of GROMACS package. SASA addresses the surface hydropathic property of the protein. SASA calculation is performed by defining the radius of a probe sphere (usually the van der Waals (vdW) radius of water) which is ‘rolled’ along the surface of the protein. If the probe overlaps with the vdW radii of the atoms of amino acids, the area is not accessible by the solvent and therefore accounted to the solvent excluded surface [33]. Intermolecular interaction study by gmx hbond provides the data about amino acids involved in stabilization of protein structure. Usually, H-bond is formed by the interaction of a hydrogen atom that is covalently bonded to an electronegative atom (donor D) with another electronegative atom (acceptor A) [34]. The acknowledged H-bond length should be less than 0.35 nm. The essential dynamics study or principal component analysis (PCA) was performed to understand the total movement of apo-protein and aptamer-bound protein. The gmx covar and gmx anaieg were utilized to diagonalize the covariance matrix and calculate the overlap between PCs and coordinates of the trajectory [35]. The free energy landscape was performed using gmx sham tool to find lower energy minima conformer.

## Results

**Fig. 1:**
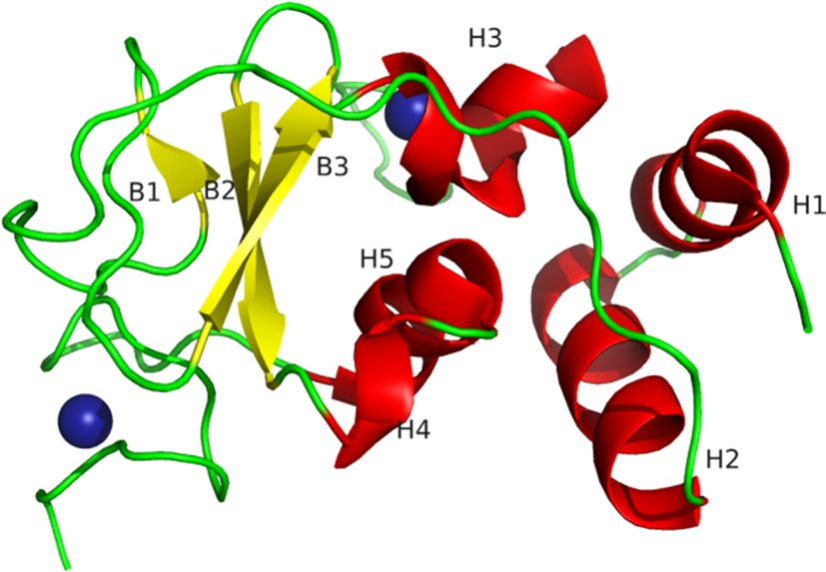
The schematic representation of 3-dimensional 3D crystal structure-NSP10 (PDB-6ZCT). H represents the alpha helices, B represents beta sheets and blue sphere indicates zinc ions

The secondary structure of NSP10 consists of 4 long α-helices and 3 β-sheets in which 2 sheets being antiparallel to each other (Fig. 1) [33]. The protein contains two Zn ions as cofactor and binds to the H3 and the loop adjacent to it (Cys73, Cys77, His83, Cys90) and the other Zn ion at the C-terminal end (Cys117, Cys120, Cys128, Cys130)—a conserved site in the coronavirus protein.

### Aptamer selection

Based on the selection criteria as discussed in the methodology, RNA aptamers were screened and docked to the NSP10. The thermodynamic parameters of the screened aptamers are listed in the Table 1. It was observed that all the 10 RNA aptamers formed secondary hairpin loop conformation with minimum of 5 Watson–Crick base pairing (Additional file 1: Figure S1). Generally, hairpin conformations are considered as the ideal conformity to limit it from fluctuations and high number of Watson–Crick base pairing preserves the aptamer conformation while docking [36]. The energy minimized stable RNA aptamers were directed for further docking studies.

**Table 1:** The sequence composition and thermodynamic properties of screened RNA aptamers

S. no	Name of RNA aptamers	Sequences (5′–3′)	GC content (%)	Minimum free energy (kcal/mol)
1	RNA-001	GGAAACGGAAUGUCGAUUUCGUGGUUAUCC	46.67	− 5.90
2	RNA-005	GGGUUUGGACUUAGAUUUGGAACCCGACCC	53.33	− 8.90
3	RNA-010	UUUGGCCAACGGGAGAGUUAAGGUCAUAAA	43.33	− 6.40
4	RNA-025	GUGAUAUCUAUGAAUGCGUAGCAGUGGCAC	46.67	− 6.60
5	RNA-040	UGGUAAUCAGUCUGGAUUGAGUCAGACCCA	46.67	− 6.20
6	RNA-053	UGUUUGUACACGACAAAAGUGUCGUGUACA	40.00	− 15.60
7	RNA-073	GAGUUUGUGUGCGGUAGCAUCAUCAAACUC	46.67	− 11.70
8	RNA-086	ACGUAAGGGGUGAUAUUGAUUACGCCGCGU	50.00	− 9.30
9	RNA-091	AAGUGAAAUGGUGUGCCGCACUGUUUGCUU	46.67	− 8.10
10	RNA-097	GUUCUCUUUACACAGUUGAGGGGAAUAAAC	40.00	− 8.30

### Protein-aptamer docking studies

The NSP10 was known to be essential for proper functioning of NSP16 and NSP 14 as it stabilizes the methyl donor binding sites [2, 37]. In SARS-CoV, the production of NSP10 was found to be 3–6 folds higher than that of NSP14 and NSP16 for simultaneous formation of NSP10-NSP14 and NSP10-NSP16 complexes. The interaction of NSP10 with NSP14 was discovered imperative, as it maintains the structural makeup of ExoNuclease (ExoN) domain and fully unleash the ExoN activity of the protein [38]. Both the methyltransferase enzymes shared few common interaction sites with NSP10 and the recurrent crucial residues of NSP10 involved in NSP10-NSP14 and NSP10-NSP16 were Lys43, Leu45, Lys93, and Tyr96 [39]. Docking was performed to analyse the binding ability of aptamers with NSP10, thereby inhibiting the NSP10-methyltransferase complex formation.

All the 10 RNA aptamers were docked against NSP10 using HADDOCK v2.2 [25]. For the data driven method, the interface residues of NSP10-NSP16 were loaded as active residues for docking. The interface residues of NSP10-NSP16 were found by analyzing the crystal structure of 6W75—PDB id using DIMPLOT of LigPlot + package [26]. In Table 2, HADDOCK score and other energies involved in scoring function of top 3 complexes with RMSD less than 2 Å are summarized.

**Table 2:** Parameters computed by HADDOCK for protein-aptamer docking

Protein-aptamer complex	Haddock score	Van der Waals energy (kcal/mol)	Electrostatic energy (kcal/mol)	Desolvation energy (kcal/mol)	Buried surface area (Å^2^)
NSP10-053	− 88.5 ± 7.0	− 65.8 ± 3.2	− 147.9 ± 26.9	− 0.8 ± 2.9	1747 ± 36.4
NSP10-001	− 87.7 ± 11.5	− 60 ± 7.0	− 190.4 ± 26.8	4.4 ± 3.1	1567 ± 106.4
NSP10-010	− 86.1 ± 12.3	− 56.8 ± 9.5	− 166.9 ± 27.9	− 0.9 ± 3.3	1677.9 ± 62.2

**Fig. 2:**
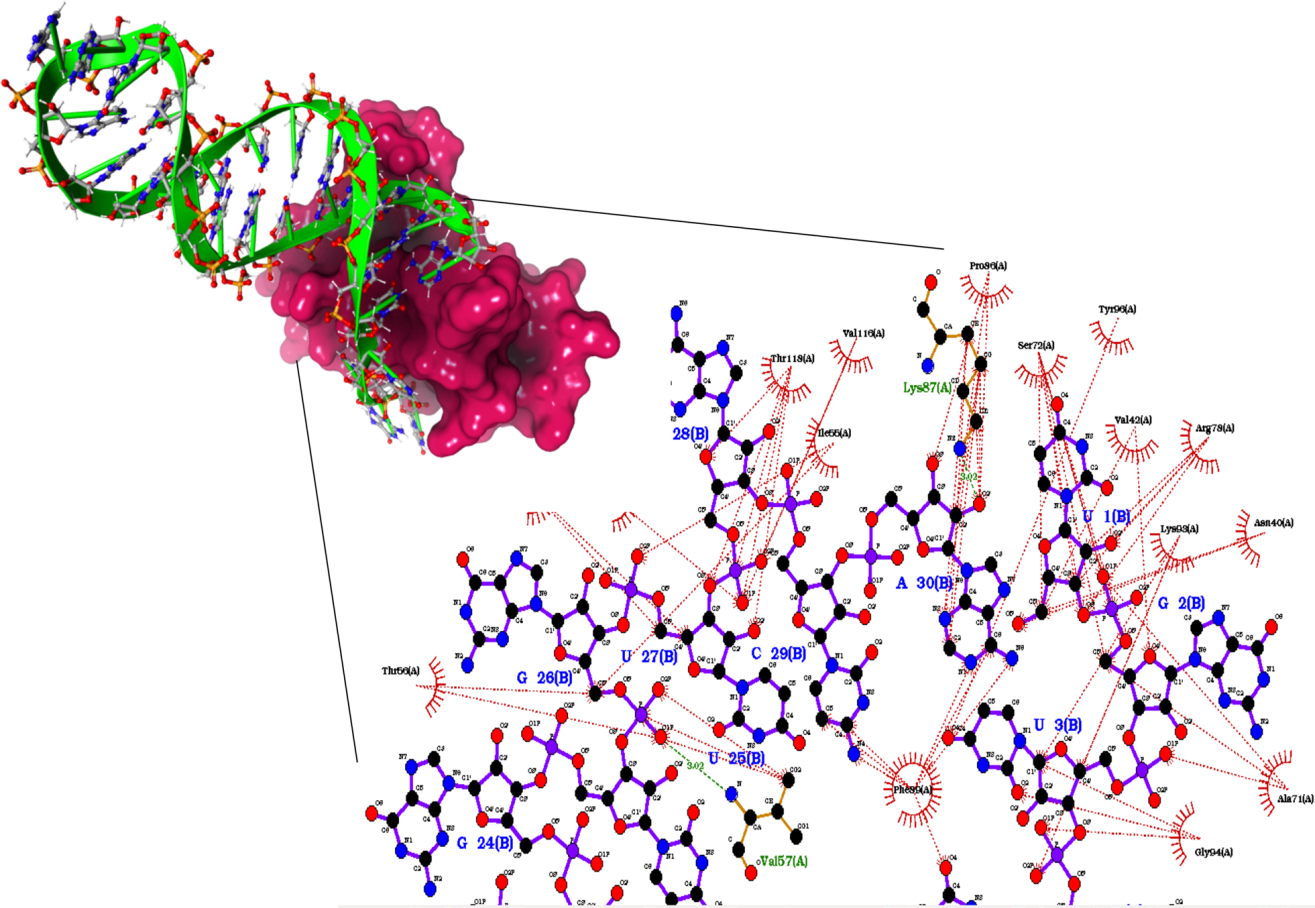
(A) The binding mode of RNA-053 aptamer with NSP10 (Ruby red surface represents NSP10 and lime green represents RNA aptamer-053. (B) 2D plot of interacting residues in NSP10-053 complex by LigPlot + (Green line represents amino acids involved in H-bonding whereas maroon represents hydrophobic interactions)

The protein-RNA interface regions were distinguished by the positively charged residues to which anionically charged nucleotides of RNA bind [40]. The H-bond analysis of NSP10-NSP16 complex exposed the involvement of residues such as Lys43, Leu45, Ala71, His80, Lys93, Gly94, Tyr96 in its binding pocket. Docking analysis revealed significant binding interactions of RNA aptamers at NSP16 binding domain in NSP10. Figure 2 illustrates the binding pose of RNA-053 with NSP10 (referred as NSP10-053). The G26, A30 nucleotides of RNA-053 formed 2 H-bonds with α-amino acids—Val57 (N atom) and Lys87 (terminal zeta N atom) at distance of 3.02 Å each.

**Fig. 3:**
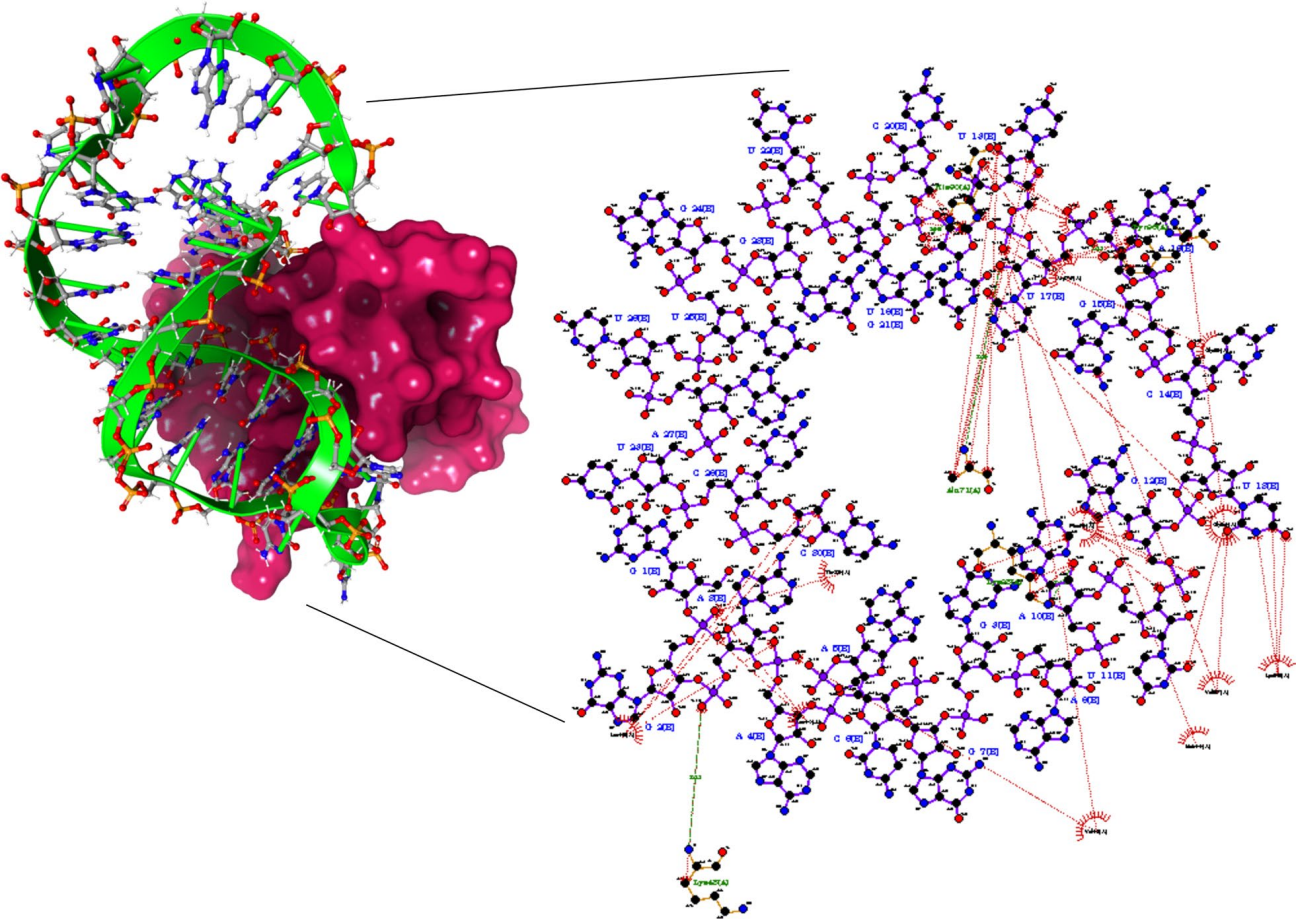
(A) Binding conformation RNA-001 aptamer with NSP10 (Ruby red surface indicates NSP10 conformation and lime green indicates RNA-001 conformation). (B) 2D graphical interpretation of binding pocket of aptamer in NSP10

The regions near hairpin loop of RNA-001- A10, A16, U17, U19 coordinated the complex by interacting with Lys93, Tyr96, Ala71, His80 at H-bond distance of 3.05 Å, 3.12 Å, 3.29 Å and 2.95 Å respectively. Besides, A3 also formed H-bond with hydrophilic Lys43 at 3.12 Å. However, the backbone phosphate groups of RNA were also involved in salt bridge formation with positively charged Arg78, His80, Lys95. The interaction between RNA-001 and NSP10 (referred as NSP10-001) is displayed in Fig. 3.

**Fig. 4:**
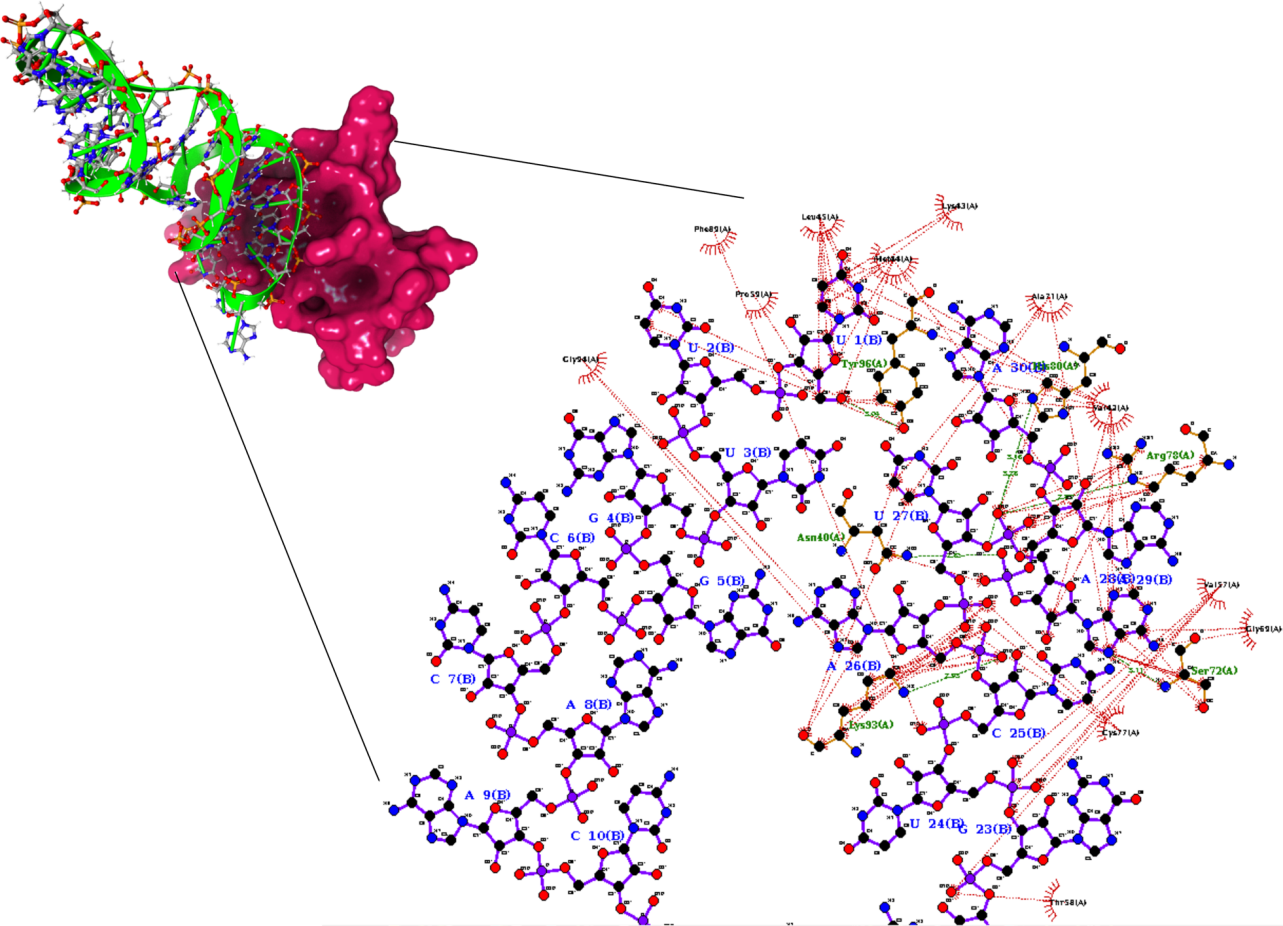
(A) Lower energy docked pose of RNA-010 at NSP10/NSP16 interface in NSP10 (Ruby red surface represents NSP10 and limegreen colour represents RNA aptamer-010). (B) Displays 2D interaction plot of NSP10-010 complex

In case of NSP10-010 complex (Fig. 4), the stem of aptamer was highly involved in hydrogen bonding with the protein. The amino acid–base interactions were Asn40 (2.85 Å)—A29, Ser72 (3.11 Å)—A29, Arg78 (2.83 Å)—28A, His80 (3.16 Å, 3.28 Å)—28A, Lys93 (2.93 Å)—C25, Tyr96 (3.04 Å)—U1. Additionally, 4 salt bridges were found between phosphate group of aptamers and Arg78 (2 salt bridges), His80, Lys93 of NSP10. Apart from hydrogen bonding in protein-aptamer complex, the hydrophobic interaction also plays a vital role in maintaining the aptamer inside the binding pocket of protein. Table 3 reveals the amino acids involved in hydrophobic bonding with the aptamers.

**Table 3:** Comparison of residues involved in hydrophobic bonding of aptamers against NSP16 binding domain of NSP10

Name of the complex	Interacting amino acids of NSP10
NSP10-NSP16 (PDB-6W75)	Asn40, Cys41, Val42, Met44, Thr47, val57, Pro59, Gly69, Gly70,Cys77, Arg78
NSP10-053	Asn40, Val42, Ile55, Thr56, Ala71, Ser72, Arg78, Pro86, Phe89, Lys93, Gly94, Tyr96, Val116, Cys117, Thr118, Gly121
NSP10-010	Val42, Lys43, Met44, Leu45, Val57, Thr58, Pro59, Gly69, Ala71, Cys77,Phe89, Gly94
NSP10-001	Thr39, Asn 40, Val42, Met44, Leu 45, Val57, Gly69, Ser72, Arg78, Phe89, Gly94, Lys95

From the molecular docking simulation, it is hypothesized that the binding of aptamers to NSP10 may inhibit the obligatory function of protein i.e., activation of methyltransferase enzymes in SARS-CoV-2. Although these aptamers were believed to retard NSP10, in order to check the stability of the docked complexes, they were subjected for molecular dynamics simulation studies over 200 ns.

### MD analysis

**Fig. 5:**
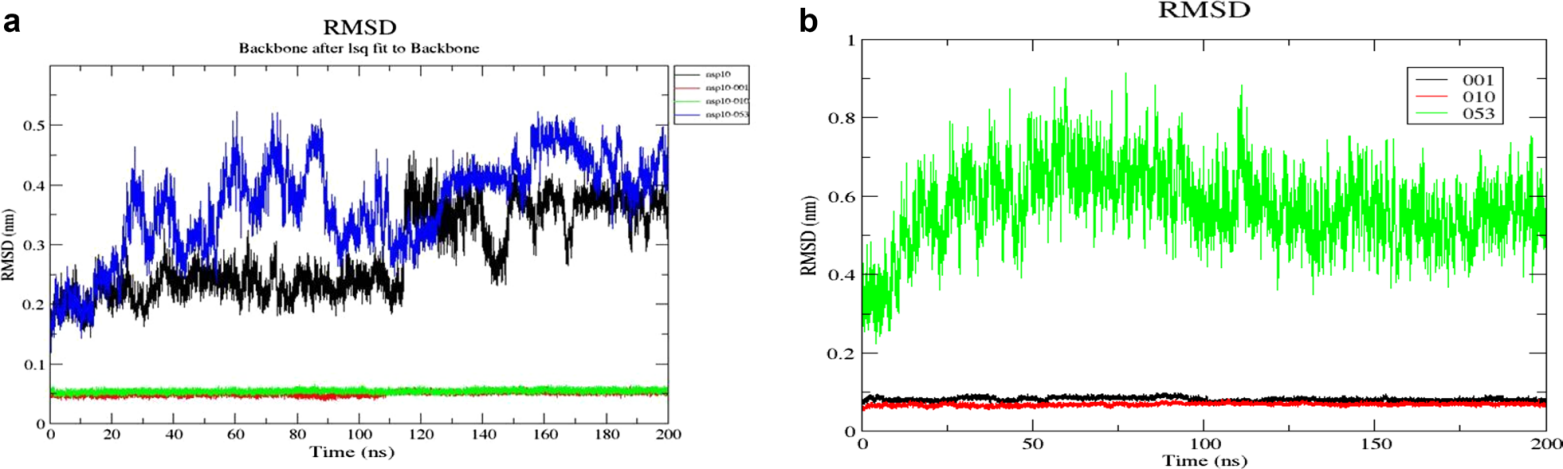
**A** RMSD plot of backbone atoms of NSP10 in free state and RNA aptamer-bound state over 200 ns simulation period. **B** RMS deviation graph of 3 aptamers in protein bound state

**Fig. 6:**
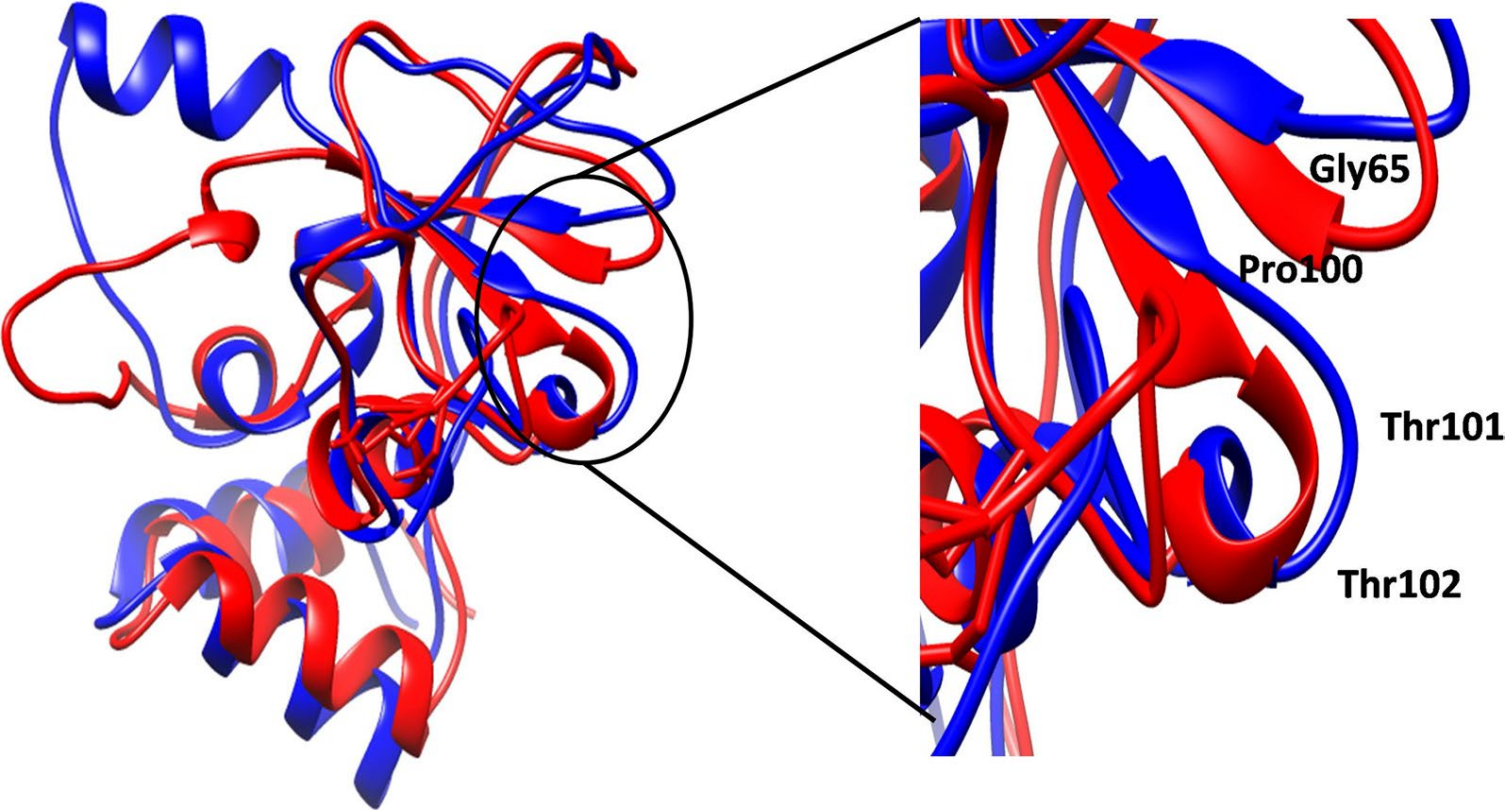
The superimposed 3D structure of simulated free state NSP10 at 0 ns (Red ribbon) and 114.5 ns (Blue ribbon) which shows the conformational changes of Gly65, Pro100, Thr101, Thr102 residues

The conformational stability of the apo-protein and protein-aptamer complexes were analysed by plotting RMSD (Fig. 5A). The average RMSD value of NSP10 was 0.282 ± 0.07 nm found between the range of 4.0 × 10^–4^–0.45 nm. The protein was stable after 40 ns of the simulation and there was a sudden spike at 114.5 ns which might be due to conformational change in Gln65 and Pro100-Thr102 of B2 and B3 into loop structure (Fig. 6). Later the protein stabilized after 150 ns with little or negligible deviations. The RMSD of NSP10-053 aptamer complex was downright, starting from the 0 to 200 ns with the mean value of 0.366 ± 0.08 nm but interestingly showed convergence from 130 to 150 ns. The backbone RMSD of NSP10-001, NSP-010 complexes were highly stable and formed plateau throughout the MD simulation with the mean value of 0.051 ± 3.4 × 10^–3^ nm and 0.054 ± 2.8 × 10^–3^ nm respectively. The NSP10-001 showed slight deviation around 75–110 ns whereas the NSP10-010 showed convergence after 1.2 ns itself. The average RMSD (Fig. 5B) of RNA aptamers 001,010, 053 were 0.082, 0.069, 0.572 nm. All the 3 aptamers were stabilized after 100 ns but lower RMSD values of 001 and 010 indicated its stronger binding with NSP10 protein.

**Fig. 7:**
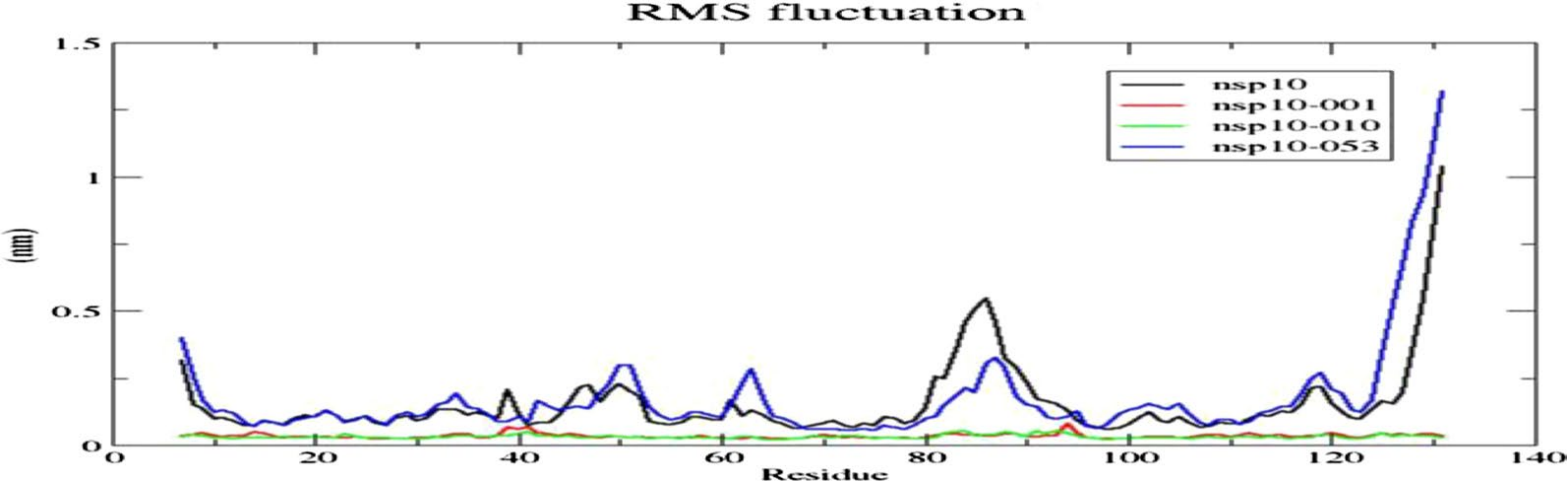
Root Mean Square Fluctuation (RMSF) plot of NSP10 with each aptamer. RMSF was calculated for Cα atoms of the protein

The residual flexibility of the protein and the docked complexes were assessed by Root Mean Square Fluctuation (RMSF) plot. From the Fig. 7, NSP10-NSP16 interface residues did not show higher fluctuation however the residues around His83—Gly88 and C-terminal ends of NSP10 showed higher fluctuation, which indicated the region contains loop. The average RMSF values of NSP10, NSP10-001 and NSP-010 were 0.15 ± 0.13 nm, 0.034 ± 0.008 nm and 0.031 ± 0.006 nm respectively. Lesser residual fluctuation of NSP10 in docked complexes manifested the binding of RNA aptamers with the protein. The strong interaction of aptamers-001,010 with His80 possibly limited the residual mobility of His83—Gly88 loop found between H3 and B3 in docked complexes. The residual flexibility of NSP10-053 complex followed similar pattern as of free state protein.

**Fig. 8:**
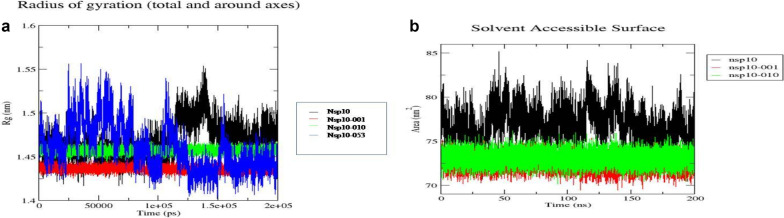
**A** Rg plot and **B** SASA plot of apo-protein (NSP10) and docked complexes (protein-aptamer) over simulation period of 200 ns

The change in compactness of unbound NSP10 and the aptamer-bound NSP10 was evaluated by Rg as a function of simulation time. The intermediate Rg values of NSP10, NSP10-001, NSP10-010 and NSP10-053 was found in between 1.4 and 1.5 nm. From the Fig. 8A, Rg plots of NSP10-001 and NSP10-010 complexes showed lesser fluctuation, thus the protein structure was found to be condensed throughout the 200 ns run. SASA analysis was performed to study about the surface area of protein that is accessible to the solvent. The mean SASA of NSP10 and the aptamer-bound protein were in the range of 72–76 nm^2^. SASA of the protein decreases with increment in compactness of the protein. From Fig. 8B, it is evident that solvent accessible surface area of aptamer-bound protein got decreased compared to free state protein. Significant change in SASA suggested the prospect of protein aggregation, which may lead to loss of functional behaviour of the protein [41].

**Fig. 9:**
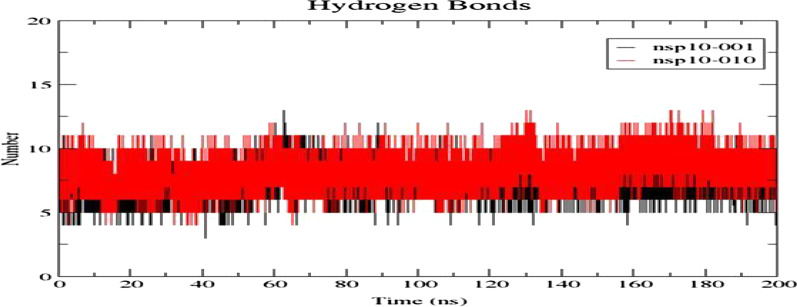
The intermolecular H-bond analysis of NSP10 with aptamers-001, 010. Both the complexes exhibited few stable H-bonds throughout the simulation

The interactions between protein-RNA aptamer complexes were assessed by g_hbond module. The hydrogen bond interaction stabilizes the protein-aptamer complex. Throughout the simulation period, at least 3–4 H-bonds were found between protein and the aptamer in both the systems (Fig. 9). Additionally, both the aptamers formed H-bond with His80 found in helix over 200 ns run. After 30 ns of MD simulation, the aptamer established constant intermolecular H-bond with the residues Ala71, Ser72, His80, Tyr96 in NSP10-010 similarly in NSP10-001 complex, after 60 ns of run the aptamer had stable interaction with Asn40, Arg78, His80, Lys93, Lys95, Tyr96 residues of NSP10.

**Fig. 10:**
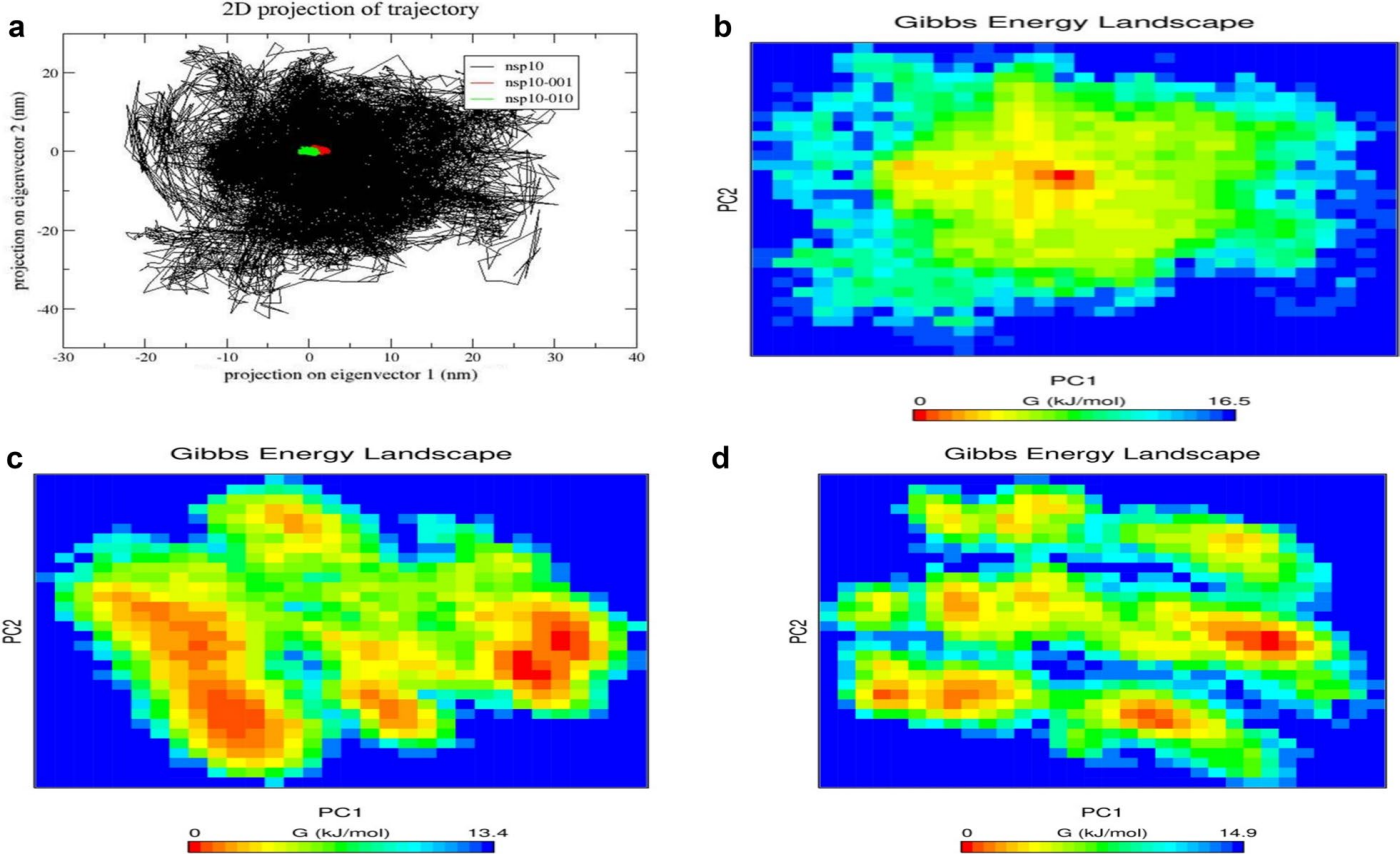
**A** Principal Component Analysis (PCA) plot of free state and RNA-001, 010 aptamers bound NSP10. The Free Energy Landscape of **B** NSP10 alone, **C** NSP10-001 complex and **D** NSP10-010 complex

The collective motion of protein was studied by essential dynamics or Principal Component Analysis (PCA). The essential dynamics study was carried out to have deeper knowledge about conformational stability and flexibility of residues. In general, the protein is required to have certain degree of rigidity, flexibility to perform its functions freely. The sturdy interaction between protein and the ligand would restrict the motion of protein, hence making the protein not to attain the required specific conformation essential for its activity. The PCA (Fig. 10A) plot corresponds that total motion of protein bound with 001, 010 aptamers showed small cluster of conformational space compared to free state protein which reflects the minimal atomic fluctuation of bound NSP10. These result correlated with other analysis like RMSD, RMSF and Rg encompassing the stability of aptamer-bound NSP10 protein. This higher rigidity of protein in docked complexes might hinder the binding of NSP16 with NSP10 resulting in partial viral RNA capping.

To examine the minimum free energy conformation ensemble of protein, the free energy landscape analysis was performed using first two PC’s i.e., PC1 and PC2. FES analysis provides Gibbs free energy difference of protein conformation in docked complexes with high accuracy. From Fig. 10B, C, it is observed that the aptamer-bound protein has low Gibbs free energy than unbound protein. This revealed that the protein had energetically favourable conformational transition when bound with aptamers—001 and 010. In both the complexes, energy basin remained connected with less energy barrier which exhibited the stable affinity between protein and RNA aptamers. From FES analysis, it is inferred that aptamers restricts the free motion of protein to a noteworthy degree and accomplished local minima compliance at various points.

## Discussion

In this study, Non-Structural Protein 10 (NSP10) has been suggested as the potential drug target in SARS-CoV-2. To date, several lead molecules have been proposed and being investigated against various targets of coronavirus. With the high exponential mutational rate of virus, the search for potent drug/lead molecules has been complicated. In this work, we have designed RNA based aptamers to act as an inhibitor against NSP10/ NSP14 and/or NSP10/NSP16 interface site. The screened aptamers were docked to NSP10. Although, RNA-073 aptamer shows better minimum free energy i.e., − 11.70 kcal/mol (2nd best) but it failed to show considerable amount of binding with the target NSP10. The work was further moved forward to study about the dynamics of best protein-aptamer complex. Considering that the protein-aptamers complex exhibits a very dynamic nature, in this study we have implemented molecular dynamics study for a time of 200 ns to mimic the natural environment. The dynamics study provided more in-depth insights into the mechanism of binding. Initial docking results revealed that the RNA-053 aptamers pose as a significant molecule among screened aptamers. Surprisingly, during the MD analysis, the above said RNA-053 aptamer moved out of the binding pocket at the end of MD simulation, signifying the best inhibitor should have optimum size thus it fits inside the pocket perfectly [42]. On comparative analysis of dynamics of apo-protein and protein-RNA aptamer complex, it is proposed that the aptamers have stronger binding with NSP10 at specific residues such as Lys43, His80, Lys93, Tyr96. The small movement of complexes in PCA plot indicated the misfolding/unfolding of NSP10. The resultant of this study is RNA-based aptamers can be synthesized and experimentally developed as a potential anti-viral molecule against SARS-CoV-2 with several clinical studies.

## Conclusions

Amidst the COVID-19 pandemic situation, there is a much demand of less toxic and highly potential drugs in the market. The distinguished non-structural protein 10 is found only in coronaviruses, shares ~ 99% of sequence identity with SARS-CoV and involved in mRNA cap methylation. Binding of this protein with NSP14 and NSP16 is essential as it increases the methyl transferring activity to certain folds in SARS-CoV-2. Computationally designed RNA aptamers were employed to disrupt the NSP10/NSP14 and NSP10/NSP16 interface, a significant factor of RNA capping mechanism. Docking studies identified the potential 3 RNA-based aptamers with high binding affinity. Atomistic model simulation revealed that among 3 chosen aptamers, 2 aptamers-001 and 010 binds with NSP10 at crucial residues Lys43, His80, Lys93, Tyr96. This interaction encourages us to believe in high chances of inhibitory action by aptamers against NSP10. Our research work greatly contributes for the discovery of potential candidate against highly infectious SARS-CoV-2. The efficacy and inhibitory concentration of designed RNA aptamers must be evaluated using in-vitro and in-vivo studies.

## Supplementary Information

**Additional file 1**. The secondary hairpin loop conformation of screened aptamers.

## Data Availability

All data used in the study is given as supplementary data.

## Abbreviations

GROMACS: GROningen MAchine for Chemical Simulations
HADDOCK: High Ambiguity Driven biomolecular DOCKing
H-bond: Hydrogen bond
MD: Molecular dynamics
NSP: Non-structural protein
ORF: Open reading frame
PCA: Principal component analysis
PDB: Protein Data Bank
Rg: Radius of gyration
RMSD: Root mean square deviation
RMSF: Root mean square fluctuation
RNA: Ribonucleic acid
SARS-CoV-2: Severe Acute Respiratory Syndrome CoronaVirus-2
SASA: Solvent accessible surface area
SELEX: Systematic Evolution of Ligands EXponential enrichment
