# Supplementary material for: Search for RNA aptamers against non-structural protein of SARS-CoV-2: Design using molecular dynamics approach
Source: Beni Suef Univ J Basic Appl Sci. 2021 Oct 12;10(1):64. doi: 10.1186/s43088-021-00152-5 (PMC8506486; doi:10.1186/s43088-021-00152-5)
Supplement: Supplementary file 1 — Additional file 1. The secondary hairpin loop conformation of screened aptamers. [file 43088_2021_152_MOESM1_ESM.docx]

**Supplementary figure:**

**
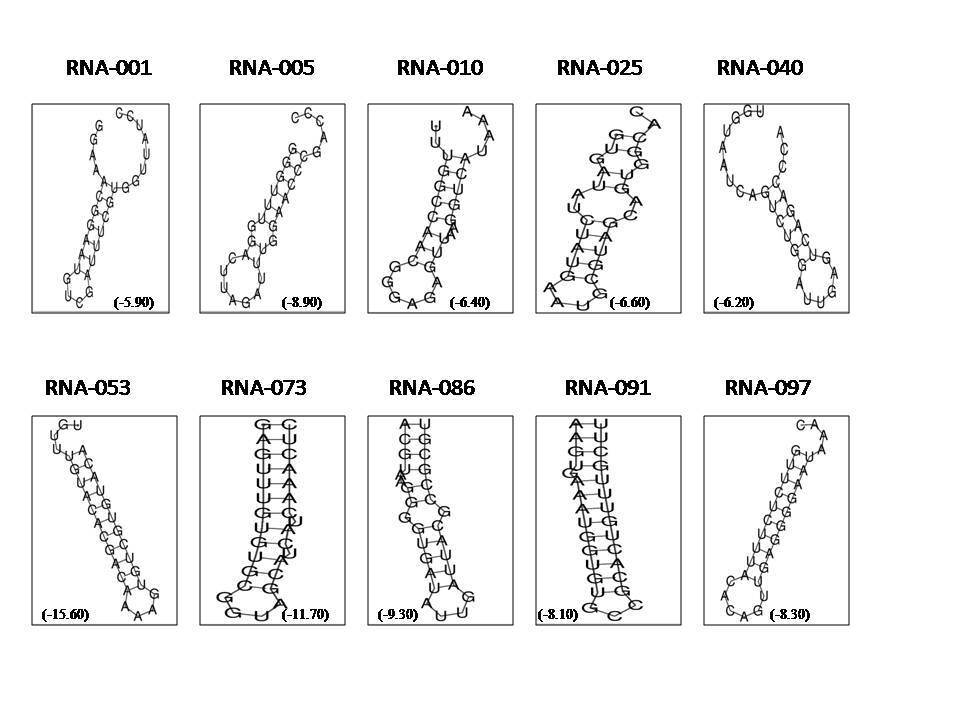
**

**Figure S1.** The secondary hairpin loop conformation of screened aptamers. The bracketed text indicates their corresponding minimum free energy (MFE) respectively.
